# The choroid plexus—a multi-role player during infectious diseases of the CNS

**DOI:** 10.3389/fncel.2015.00080

**Published:** 2015-03-12

**Authors:** Christian Schwerk, Tobias Tenenbaum, Kwang Sik Kim, Horst Schroten

**Affiliations:** ^1^Department of Pediatrics, Pediatric Infectious Diseases, Medical Faculty Mannheim, Heidelberg UniversityMannheim, Germany; ^2^Division of Pediatric Infectious Diseases, Johns Hopkins University School of MedicineBaltimore, MD, USA

**Keywords:** blood-brain barrier, blood-cerebrospinal fluid barrier, central nervous system infection and inflammation, choroid plexus, pathogen

## Abstract

The choroid plexus (CP) is the source of cerebrospinal fluid (CSF) production and location of the blood-CSF barrier (BCSFB), which is constituted by the epithelial cells of the CP. Several infectious pathogens including viruses, bacteria, fungi and parasites cross the BCSFB to enter the central nervous system (CNS), ultimately leading to inflammatory infectious diseases like meningitis and meningoencephalitis. The CP responds to this challenge by the production of chemokines and cytokines as well as alterations of the barrier function of the BCSFB. During the course of CNS infectious disease host immune cells enter the CNS, eventually contributing to the cellular damage caused by the disease. Additional complications, which are in certain cases caused by choroid plexitis, can arise due to the response of the CP to the pathogens. In this review we will give an overview on the multiple functions of the CP during brain infections highlighting the CP as a multi-role player during infectious diseases of the CNS. In this context the importance of tools for investigation of these CP functions and a possible suitability of the CP as therapeutic target will be discussed.

## The Choroid Plexus and the Blood-Cerebrospinal Fluid Barrier

First evidence for the existence of barriers between the blood and the central nervous system (CNS) was found around the beginning of the 20th century, when Paul Ehrlich and his associate Edwin Goldman demonstrated that dyes injected into the vascular system were taken up by all organs except the brain and the spinal cord, whereas, when applied directly into the cerebrospinal fluid (CSF), stained only the brain tissue (Ehrlich, 1885, 1902; Goldman, 1909). These pioneering experiments eventually lead to the discovery of the blood-brain barrier (BBB) located at the endothelial cells of the brain capillaries, which are connected to each other by tight junctions (TJs) and supported by additional cells like astrocytes and pericytes. The BBB constitutes the largest interface for exchange between the blood and the brain, and provides a barrier between the blood and the interstitial fluid (ISF) of the brain parenchyma (Abbott et al., 2010). Importantly and in contrast to the brain parenchyma, the choroid plexus (CP), a highly vascularized endothelial-epithelial convolute in the ventricular system, was stained when dyes were injected into the blood but not subsequent to injection of the dyes into the CSF, pointing to a second barrier located at the epithelium of the CP, which is termed blood-cerebrospinal fluid barrier (BCSFB).

The main function of the CP is the production of the CSF. Overall four *plexus choroidei* exist, which are located in the two lateral ventricles and the third and the fourth ventricle of the brain. In the CP the endothelial cells are fenestrated, a feature which is required for the production of CSF from the blood, and therefore do not significantly contribute to barrier function (Wolburg and Paulus, 2010). In contrast, to enable shielding of the CSF from the blood, the CP epithelial cells are interconnected by dense TJ strands. Additionally, the CP epithelium is characterized by the presence of specific transporter systems and a low pinocytotic activity, thereby regulating the crossing of substances. Therefore, the epithelial cells of the CP are the morphological correlate of the BCSFB (Spector and Johanson, 1989; Strazielle and Ghersi-Egea, 2000; Wolburg and Paulus, 2010), and the BCSFB presents a barrier between the blood and the ventricular CSF (Abbott et al., 2010). There is no equivalent barrier between the CSF and the brain parenchyma in adult mammals, although a transitory barrier was observed in the developing sheep brain formed by so-called “strap” junctions connecting the neuroependymal cells lining the ventricular wall (Møllgård et al., 1987; Wolburg and Paulus, 2010; Liddelow, 2011). The ISF in the brain parenchyma and the CSF are, however, not identical, even though they are free to communicate at several locations and the ISF contributes significantly to the CSF (Abbott, 2004; Abbott et al., 2010). The ependymal cell layer as well as the pia mater and glia limitans, the latter two separating the brain parenchyma from the CSF in the subarachnoidal space, could be considered as less tight “cellular barriers” compared to the TJ-based “molecular barrier” located at the epithelial cells of the CP.

## Tools for Investigation of Choroid Plexus Functions During Infectious Disease

A detailed analysis of putative roles of the CP during infectious diseases of the CNS implicates the need for tools to investigate pathogenesis executed at the CP during the course of disease. Besides animal models, *in vitro* systems of the BCSFB, which faithfully mimic the characteristic *in vivo* properties of this barrier including a good barrier function, are of special importance. Several BCSFB *in vitro* models based on primary cells or cell lines have been described (Strazielle and Ghersi-Egea, 2011). Functional *in vitro* models are of importance for analysis of the mechanisms of host-pathogen interaction as well as transmigration of immune cells, but also to study candidate drugs destined for treatment of CNS infections.

Since the morphological correlates of the BCSFB are the epithelial cells of the CP, the current *in vitro* models of the BCSFB are based on this cell type. For primary models CP epithelial cells have been isolated from diverse animal species including cattle, goat, pig and rodents (Strazielle and Ghersi-Egea, 2011). A primary model system with excellent barrier function is described with primary porcine CP epithelial cells (PCPEC). PCPEC display a polar epitheloid morphology with continuous TJs and develop a high TEER when grown on filter supports. Additionally, PCPEC display active transport properties (Gath et al., 1997; Haselbach et al., 2001).

Although *in vitro* models based on cell lines are more prone to exhibiting characteristics that differ from the *in vivo* situation than primary models, they provide the advantages of easier handling and accessibility to molecular manipulation. Several cell lines with origin of the CP epithelium have been introduced, but many of them present only limited barrier properties (Strazielle and Ghersi-Egea, 2011). A porcine model based on an immortal cell line generated from PCPEC with good barrier function has been described, but not yet used for studies with infectious pathogens (Schroten et al., 2012). Furthermore, the human CP epithelial papilloma cell line HIBCPP has been established as functional model of the BCSFB. HIBCPP cells display a good barrier function as well as apical/basolateral polarity (Ishiwata et al., 2005; Schwerk et al., 2012; Gründler et al., 2013).

## The Choroid Plexus as Entry Site for Pathogens

To cause disease in the CNS pathogens must cross one of the BBBs. Access of pathogens to the brain via the BBB is well described (Kim, 2008; Pulzova et al., 2009), but there is also definitive evidence for the CP and the BCSFB as entry site of pathogens into the CNS (Dando et al., 2014). To cross cellular barriers pathogens can employ different strategies. They can either exploit a transcellular penetration through the cell or they may achieve opening of TJs for a paracellular mechanism. Additionally, pathogens can also hijack infected phagocytic host cells and penetrate into the CNS via a “Trojan horse” strategy (Kim, 2008; Pulzova et al., 2009; Dando et al., 2014). Following crossing of these barriers the pathogens can elicit inflammatory reactions in the brain, ultimately leading to diseases like meningitis and meningoencephalitis.

**Figure 1:**
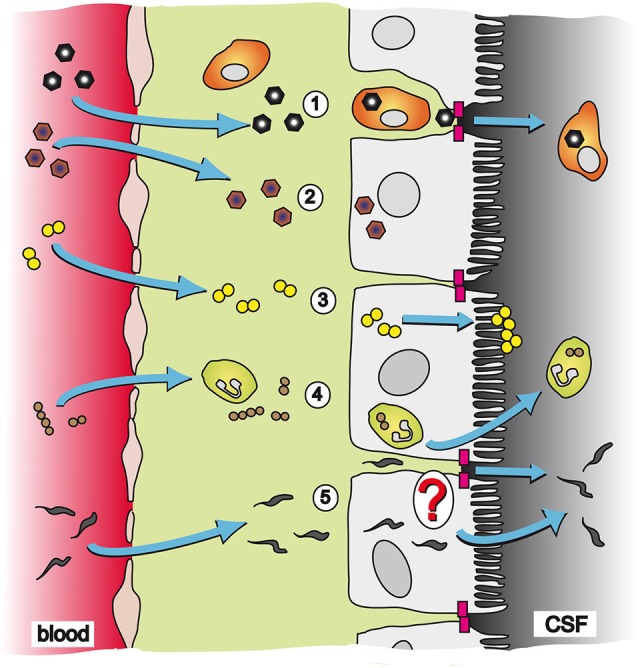
**Pathways and strategies of pathogen invasion into the CNS via the CP**. The CP is an endothelial-epithelial convolute responsible for production of the CSF. Whereas the blood capillaries in the CP are fenestrated, the epithelial cells of the CP are connected to each other by TJs (magenta blocks) and present the morphological correlate of the BCSFB. (1) CVB3 has been described to cross the BCSFB inside of a specific population of myeloid cells, which is highly susceptible to infection upon traversal of the CP epithelial TJs; (2) EV30 can invade into CP epithelial cells and replicate inside the host cell; (3) The bacterium *N. meningitidis* can directly invade into CP epithelial cells. After transmigration through the cell layer *N. meningitidis* forms microcolonies at the apical cell side; (4) *S. suis* has been shown to invade into PMNs and crosses the BCSFB in a transcellular fashion employing a “Trojan horse” strategy; and (5) The parasite *T. b. brucei* can transmigrate through the CP epithelium to enter the CSF. The exact mechanism of translocation by *T. b. brucei* is not clear yet. It is hypothesized that the parasite follows a paracellular route after opening of TJs. Alternatively, *T. b. brucei* could directly invade CP epithelial cells for a transcellular mechanism.

Microorganisms known to use the BCSFB to reach the CNS belong to different groups of pathogens including viruses, bacteria, fungi and parasites. In the following we provide examples for selective pathogens for which the CP as entry site into the brain has been described. An overview of the different organisms and their strategies of entry into the CNS is schematically presented in Figure 1.

### Viruses

Of the two major types of meningitis, viral and bacterial, viral meningitis occurs with an almost three-fold greater frequency compared to bacterial meningitis (Rantakallio et al., 1986; Giorgi Rossi et al., 2009). CNS infections can be caused by several distinct types of viruses, but viruses of the genus Enterovirus, part of the family *Picornaviridae*, are responsible for many cases of aseptic meningitis and encephalitis in neonates and young children. Whereas Coxsackievirus B3 (CVB3) primarily targets neonates, Echovirus 30 (EV30) causes disease mainly in infants and young children (Rhoades et al., 2011). Using a recombinant CVB3 expressing enhanced green fluorescent protein (eGFP) Feuer and co-workers analyzed CNS tropism and pathology in neonatal mice. Interestingly, at early time points high levels of virus-produced eGFP was found in the CP and further investigations in this model confirmed a role of the CP in the pathogenesis of CVB3-caused meningitis (Feuer et al., 2003; Tabor-Godwin et al., 2010). A novel population of peripherally recruited myeloid cells was found to be infected during their passage through the CP. These cells were highly susceptible to infection upon traversal of the CP epithelial TJs, although the CP epithelial cells seemed to be omitted from infection by CVB3. The authors suggest that binding of TJ-located coxsackie and adenovirus receptor (CAR) by CVB3 virions assist in dissemination of the virus (Tabor-Godwin et al., 2010). In contrast, the *in vitro* studies with the human BBB endothelium revealed that CVB and poliovirus (PV) hijack the host cell signaling pathways for infection of the BBB. PV enters the BBB endothelium by dynamin-dependent caveolar endocytosis, and entry depends on intracellular signals triggered by virus attachment to PV receptor, such as SHP-2, a protein tyrosine phosphatase, while calcium signaling is involved in CVB entry into the BBB (Coyne et al., 2007, 2011).

The lymphocytic choriomeningitis virus (LCMV), a rodent-borne Arenavirus, was found inside of CP epithelial cells in the same murine model as that used for CVB3 (Puccini et al., 2014). Invasion of CP epithelial cells was also shown for EV30 in a functional *in vitro* model of the human BCSFB based on HIBCPP cells (Ishiwata et al., 2005; Schneider et al., 2012). Following infection the virus replicates inside of the HIBCPP cells (Schneider et al., 2012).

The CP has also been suggested to play an important role during the development of encephalitis caused by lentiviruses. Studies have shown that the CP is infected in humans with human immunodeficiency virus (HIV), and these results are consistent with the hypothesis of the CP as a reservoir for HIV infection in the CNS. Furthermore, the CP may function as a site of hematogeneous dissemination of the virus to the brain (Falangola et al., 1995; Petito et al., 1999). Infection of the CP has also been demonstrated for Simian Immunodeficiency Virus (SIV)-infected macaques (Lackner et al., 1991; Czub et al., 1996) and Feline Immunodeficiency Virus (FIV)-infected cats (Bragg et al., 2002), and upon FIV infection CP functions are impaired (Ryan et al., 2005).

### Bacteria

Evidence for CNS entry via the CP has been provided for several bacteria. These bacteria often colonize the mucous epithelia of the upper respiratory or the intestinal tract in healthy hosts (carrier state), but also can cross these barriers to enter the blood stream and disseminate inside the host.

The Gram-negative encapsulated bacterium *Haemophilus influenzae* (*H. influenzae*) can colonize the upper respiratory tract of children. *H. influenza* type b (Hib) had been a worldwide major cause of bacterial meningitis in infants and children in the past, but routine immunization with Hib conjugate vaccines has largely eradicated Hib meningitis in many high-income countries (Kim, 2010). Experiments in infant primates have shown that Hib invades the CNS via the CP, where the earliest histopathologic lesions can be detected. Necropsied animals displayed mild-to-moderate fibrinopurulent leptomeningitis, inflammatory cells within the ventricles, and a mild stromal choroid plexitis (Daum et al., 1978; Smith, 1987).

The Gram-positive agent *Streptococcus suis* (*S. suis*) typically colonizes the tonsils of its natural host organisms, which are pigs. *S. suis* can cause several diseases in pigs, including meningitis, sepsis and endocarditis (Staats et al., 1997). Histopathological examinations in pigs have provided evidence for the BCSFB as entry point of *S. suis* into the CSF (Sanford, 1987; Williams and Blakemore, 1990; Madsen et al., 2002), and the CP was described as entry site in a mouse model as well (Domínguez-Punaro et al., 2007). Furthermore, in an *in vitro* model of the BCSFB based on PCPEC *S. suis* did invade into the host cells in a polar fashion from the physiologically relevant basolateral side, supporting a predominantly transcellular passage of the bacteria through the PCPEC layer. Interestingly, some transmigration of bacteria occurred from the apical to the basolateral side in the absence of bacterial invasion, suggesting a “reseeding” process of the blood from the brain via a paracellular pathway (Tenenbaum et al., 2009). When porcine polymorphonuclear neutrophils (PMNs) were added to this model system, some PMNs contained bacteria during the transmigration process, supporting a possible “Trojan horse” mechanism (Wewer et al., 2011). As zoonotic pathogen *S. suis* can also cause meningitis in humans (Arends and Zanen, 1988; Wisselink et al., 2000), and *S. suis* is an important emerging pathogen in man especially in Asian countries (Segura et al., 2014a,b). Importantly, employing HIBCPP cells, the polar invasion of *S. suis* into CP epithelial cells was recapitulated in a human model system (Schwerk et al., 2012).

**Figure 2:**
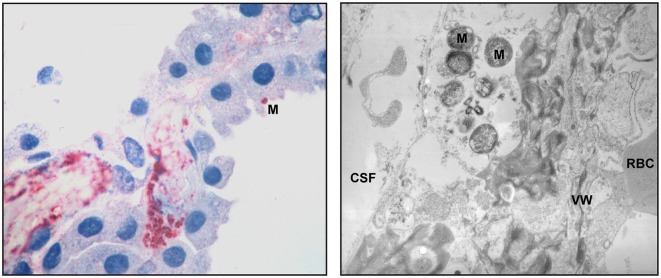
**Pathologic findings in the CP of human patients with meningococcal disease**. An immunohistochemical examination (left panel) shows abundant bacteria and bacterial antigens in the lumen of a thrombosed blood vessel and in the interstitial tissue. One *N. meningitidis* organism (M) can be seen in the surface of a CP epithelial cells. Transmission electron microscopy (right panel) demonstrates meningococci (M) in the interstitial space. The blood vessel wall (VW), a red blood cell (RBC) and the space containing cerebrospinal fluid (CSF) are pointed out. The pictures are reproduced with friendly permission from Guarner et al. (2004).

Another organism colonizing the upper respiratory tract is the human-specific Gram-negative bacterium *Neisseria meningitidis* (*N. meningitidis*). The mechanisms of CNS invasion of *N. meningitidis* are under discussion. Considered as brain entry site are the postcapillary venules and veins in the subpial and subarachnoidal spaces (Christodoulides et al., 2002; Join-Lambert et al., 2010). Evidence for the CP as gateway has been gathered from histopathological examinations of patients with meningococcal disease, where larger amounts of bacteria were found in the vessels as well as in the epithelium of the CP (Pron et al., 1997; Guarner et al., 2004), and *N. meningitidis* invades HIBCPP cells from the basolateral side *in vitro*. In the latter studies transcellularly migrated bacteria could be found at the apical membrane of the HIBCPP cells, where they did form microcolonies (Schwerk et al., 2012). Figure 2 shows pathologic findings in the CP of human patients with meningococcal disease (Guarner et al., 2004).

An important colonizer of the intestinal tract is *Escherichia coli* (*E. coli*), a Gram-negative rod-shaped bacterium commonly found in the lower intestine. Whereas extensive data exist demonstrating that *E. coli* cross the BBB, as shown by *E. coli* entry in the cerebral capillaries, not in the CP in experimental hematogenous *E. coli* meningitis (Kim et al., 1992, 2005; Kim, 2002), there is suggestion that the BCSFB may serve as an entry site. Early experiments showed that a recombinant *E. coli* expressing S fimbriae adhered to the cerebral vascular endothelium as well as the epithelial lining of the CP and ventricles of cryostat sections from rat brains (Parkkinen et al., 1988). Subsequent studies with meningitis-causing *E. coli*, however, showed that S fimbriae did not play a major role in penetration into the brain in experimental hematogenous meningitis (Wang et al., 2004).

The facultatively intracellular Gram-positive bacterium *Listeria monocytogenes* (*L. monocytogenes*) is the cause of listeriosis, a disease with a mortality rate of up to 30% in humans. As a food-borne pathogen *L. monocytogenes* typically enters the host by crossing the intestinal epithelium. *In vivo* studies have suggested that *L. monocytogenes* invades the CP including the CP epithelial cells and is also found in the CSF following infection. Bacteria were often found inside of CNS-invading mononuclear cells, pointing to an involvement of a “Trojan horse” mechanism (Prats et al., 1992; Berche, 1995; Schlüter et al., 1996). Further *in vitro* experiments showed that *L. monocytogenes* can invade sheep CP cells and HIBCPP cells. Whereas adhesion to and entry into sheep CP cells involved the *L. monocytogenes* immunogenic surface protein IspC, invasion of HIBCPP cells required action of the two listerial surface proteins Internalin (InlA) and InlB (Wang and Lin, 2008; Gründler et al., 2013). Invasion into HIBCPP cells was only observed from the basolateral cell side. Accordingly, HIBCPP cells express the host cell receptors of InlA and InlB, which are E-cadherin and Met, respectively, only on their basolateral membrane compartments (Gründler et al., 2013).

### Fungi

The most common fungal infection in the CNS is caused by *Cryptococcus neoformans* (*C*. *neoformans*), especially in regions with a high prevalence of HIV-1 or in otherwise immunosuppressed individuals (Liu et al., 2012). Although generally invasion into the brain is thought to proceed across the BBB without involvement of the CP (Chang et al., 2004; Olszewski et al., 2004; Charlier et al., 2005), rare cases of infection presenting cryptococcal choroid plexitis have been described (Cho et al., 1998; Kovoor et al., 2002; Graciela Agar et al., 2009; Kumari et al., 2010). The BCSFB, therefore, may play a minor role in CNS invasion by fungal pathogens.

### Parasites

The BCSFB is also of relevance for entrance of certain parasites into the CSF. An important example are African trypanosomes, since it has been shown in rodent models that *Trypanosoma brucei* (*T. b. brucei*) passes the fenestrated CP epithelium in early stages of infection (Masocha and Kristensson, 2012). It is hypothesized that induction of cytokine expression, including tumor necrosis factor (TNF) α, leads to opening of the BCSFB localized at the CP epithelial cells and passage of trypanosomes into the CSF (Quan et al., 1999a; Masocha and Kristensson, 2012). Strong evidence for the CP as entry site for trypanosomes into the brain was provided by electron microscopy studies, where the parasite was found to enter the CP, but was not detected in the brain parenchyma outside of blood capillaries (Wolburg et al., 2012). The parasite infection in the CSF is oscillating, with a 1 day delay to the described oscillation of trypanosome in blood, indicating a repeated crossing of the BSCFB: After entry of the CSF, which seems to be a rather hostile environment for the parasite, the trypanosomes were found to move between the cell layers of the pia mater including the Virchow-Robin space (Wolburg et al., 2012; Mogk et al., 2014a,b).

In patients with acquired immunodeficiency syndrome (AIDS) *Toxoplasma gondii* tachyzoites were found in the CP (Falangola and Petito, 1993). Other parasites involving the CP during their pathogenesis include Schistosoma species, which can move to the CP, where they shed eggs into the CNS, and *Toxocara canis*, which was found in the CP following oral administration of embryonated eggs (Masocha and Kristensson, 2012).

## The Cellular Response of the Choroid Plexus to Infection

Specialized receptors of the innate immune system, termed pattern recognition receptors (PRRs), recognize characteristic features of infectious microorganisms, the pathogen-associated molecular patterns (PAMPs). Binding of PRRs to PAMPs leads to activation of intracellular host cell signaling cascades, which culminate in the regulation of inflammatory response genes. As consequence, the host cells express *inter alia* specific cytokines and chemokines, which are involved in regulating the inflammatory response to the infecting organism (Beutler, 2009). An important group of PRRs is the Toll-like receptor (TLR) family, which are structurally characterized by extracellular leucine-rich repeats and a cytoplasmatic Toll/Interleukin-1 receptor (TIR) domain (Akira et al., 2006; Kawai and Akira, 2010). The expression pattern of TLRs in the brain, especially in the CP, has been mainly investigated in mice and rats (Laflamme and Rivest, 2001; Laflamme et al., 2003; Chakravarty and Herkenham, 2005; Rivest, 2009; Stridh et al., 2013). A recent study has examined the profile of TLR mRNA expression in the CP of adult ewes (Skipor et al., 2015), but little is known about their expression in the human brain. The TLR expression pattern of HIBCPP cells has recently been determined. Several TLRs (TLR1, 2, 3, 5, 6 and 10) were shown to be significantly expressed at mRNA level, but others (TLR4, 7, 8 and 9) exhibited no or only very little expression (Borkowski et al., 2014). It has to be kept in mind that due to their origin from a CP papilloma HIBCPP cells may not faithfully reflect the *in vivo* situation.

The cellular response of the CP to infectious pathogens points to a contribution of the CP to the host immune response. Challenge with TLR ligands including lipopolysaccharide (LPS), a ligand for TLR4, elicits the up-regulation of cytokines in the CP, as demonstrated by *in situ* hybridization of brain sections or microarray analyses in animal models (Tarlow et al., 1993; Vallières and Rivest, 1997; Nadeau and Rivest, 1999; Quan et al., 1999b; Tonelli et al., 2003; Stridh et al., 2013). Also, rats infected with *T. b. brucei* displayed expression of cytokines in the CP, an observation that agrees with the suggestion of the CP as trypanosomal brain entry site (Quan et al., 1999a). *In vitro* studies employing infection of PCPEC with *S. suis* have confirmed that CP epithelial cells are able to respond to bacterial challenge with the regulation of several genes. Among activated genes were several cytokines and chemokines including interleukin (IL) 6, IL8 and TNFα (Tenenbaum et al., 2008; Schwerk et al., 2011). Other regulated genes identified were involved in programmed cell death, which is in agreement with the observations that *S. suis* induces apoptotic pathways in PCPEC, and that TNFα causes alterations of barrier functions by cell death processes in this model system (Tenenbaum et al., 2006; Zeni et al., 2007; Schwerk et al., 2010). A compromised PCPEC barrier function could be observed after apical infection with *S. suis* (Tenenbaum et al., 2005, 2008), indicating a possible mechanism involving a paracellular “reseeding” from the CSF to the blood.

Production of chemokines and cytokines was also observed in HIBCPP cells in response to various pathogens, as exemplified by the secretion of CXCL1-3, IL8 and CCL5 following infection with EV30 (Schneider et al., 2012). Following infection of HIBCPP cells with *N. meningitidis* cytokines and chemokines such as CXCL1-3, IL6, IL8, TNFα, G-CSF and GM-CSF or IL-1β, IL6, MIP-1α, MIP-1β, CXCL1-3, MCP1 and IL8 were secreted after infection with bacteria or in experiments involving PMN transmigration after bacterial infection, respectively (Steinmann et al., 2013; Borkowski et al., 2014). In this experimental model system, the only marginally expressed TLR4 did not seem to play a significant role. The response to specific agonists indicated that TLR2/TLR6, rather than TLR2/TLR1, is involved in the cellular reaction of HIBCPP cells following infection with *N. meningitidis*. Detailed analyses pointed to a NFκB-mediated pro-inflammatory immune response involving up-regulation of the transcription factor IκBζ (Borkowski et al., 2014). The marginal expression of and response to TLR4 in HIBCPP cells is in contrast to the above-mentioned observations of proper expression and response to TLR4 made in the CP of rodents (Chakravarty and Herkenham, 2005; Rivest, 2009). It is not clear whether this discrepancy is due to species specificity or differences in experimental models, e.g., the use of the papilloma-derived HIBCPP cell line. Certainly, more experimentation is required to clarify the role of specific TLRs in the human CP.

Up-regulation of additional genes relevant to meningitis has been described in BCSFB *in vitro* models following infection with pathogens. These include cell adhesion molecules like intercellular adhesion molecule (ICAM) 1 and vascular cell adhesion molecule (VCAM) 1 (Schwerk et al., 2011; Wewer et al., 2011; Borkowski et al., 2014). In agreement, peripheral treatment with LPS caused up-regulation of genes for cell adhesion molecules in the CP in a mouse model (Marques et al., 2009). Down-regulated genes in this approach included TJ components like claudins. Interestingly, alteration of TJ morphology and protein expression, including claudin-1, was demonstrated in PCPEC infected with *S. suis* (Tenenbaum et al., 2008). Also, *S. suis* reduced the expression of claudin-2 in PCPEC (Schwerk et al., 2011), although this reduction might have a protective function, since claudin-2 can induce cation selective channels in epithelial cells (Amasheh et al., 2002).

## The Choroid Plexus as a Route for Immune Cell Entry into the CNS

The production of cytokines and chemokines by the CP epithelium, the up-regulation of cell adhesion molecules, as well as breakdown of the BCSFB can contribute to another important attribute of the CNS infectious disease, the recruitment of immune cells to the CNS. Cells of the immune system have been found in the CSF during the course of CNS infections by diverse pathogens, and the CP has been described as a gateway for immune cells trafficking into the CSF (Wilson et al., 2010; Meeker et al., 2012b). It is well known that interactions of immune cell integrins with cell adhesion molecules on the surface of CP epithelial cells play a major role during leukocyte invasion via the CP following CNS inflammation (Engelhardt et al., 2001; Meeker et al., 2012b). The first line of immune defense during CNS infection is offered PMNs, which are followed by infiltration of monocyte representing the second wave of host inflammatory response (Zhou et al., 2003). The results of the lumbar punctures in viral and bacterial meningitis indicate that during early stage of bacterial meningitis the immune response is dominated by PMNs, whereas viral CNS infections seem to be associated with lymphocytes and monocytes (Lucht et al., 1992). In this regard, the BCSFB has been shown to support entry of T lymphocytes in response to viral infection (Ryan et al., 2005).

**Figure 3:**
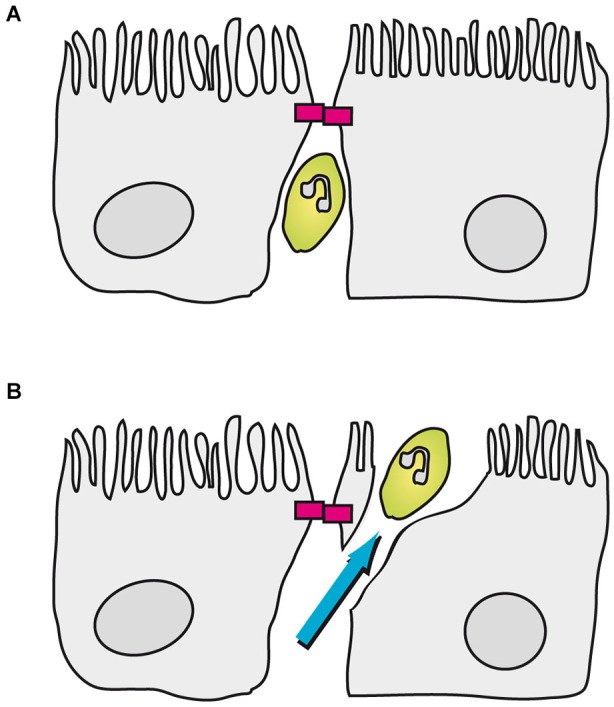
**Transmigration of porcine PMNs in an *in vitro* model of the BCSFB based on PCPEC**. In this model PMNs can be found between CP epithelial cells up to the apically located TJs (magenta blocks). **(A)** Once a PMN encounters a closed TJ, the neutrophil stops in front of the TJ. **(B)** Subsequently, the PMN finishes transmigration in a transcellular fashion, which involves the formation of a “funnel-like” structure originating from the apical membrane of the PCPEC.

Transmigration of immune cells through CP epithelial cells has been analyzed *in vitro* in porcine and human model systems of the BCSFB employing PCPEC and HIBCPP cells, respectively (Tenenbaum et al., 2013). An important question to be asked was whether immune cells exploit a paracellular or a transcellular mechanism to enter the brain. As shown by Wewer and co-workers in the porcine system following infection with *S. suis*, PMNs seem to preferentially cross PCPEC via the transcellular route. Paracellularly migrating PMNs did stop just before the apically located TJs and subsequently finished crossing of the PCPEC monolayer via employing a final transcellular step, involving the formation of “funnel-like” structures originating from the apical membrane of PECPEC (Wewer et al., 2011; Figure 3). Transmigration of PMNs through PCPEC was dependent on the integrin CD11b/CD18, an observation which correlates with the up-regulation of ICAM1 and VCAM1 in the same assay (Wewer et al., 2011). In this regard, for neutrophil migration through intestinal epithelial cells CD11b/CD18-dependent as well as—independent mechanisms have been proposed (Parkos et al., 1991; Blake et al., 2004). Further research is required to elucidate the role of cellular surface proteins during immune cell transmigration at the BCSFB.

A transcellular migration of PMNs was also detected using HIBCPP cells infected with the human-specific pathogen *N. meningitidis*, but additionally evidence for a paracellular route was found (Steinmann et al., 2013). In this model system transmigration of monocytes was only enhanced in co-culture experiments with PMNs, suggesting the notion that PMNs promote monocytes infiltration into the CNS during viral-induced encephalitis (Zhou et al., 2003). Using an *in vitro* model based on feline CP epithelial cells as well as explant cultures of feline CP epithelium, Meeker and co-workers showed that the CP epithelium supports both macrophage and peripheral blood mononuclear cells (PBMC) trafficking, which was enhanced by FIV (Meeker et al., 2012a). Since viral CNS infection was reported to support entry of T cells into the brain, T lymphocyte migration across HIBCPP cells was analyzed subsequent to infection with EV30, but only a minor effect on T cell transmigration could be observed in this system (Schneider et al., 2012).

## Complications of the CNS Infection Involving the Choroid Plexus

Processes at the CP can have a significant influence on the progress and outcome of infectious diseases in the brain. The cellular damage observed from bacterial meningitis is caused by an interplay of bacterial and host driven toxicity, with an important role of host immune cells entering the CNS (Weber and Tuomanen, 2007). Therefore, the above-mentioned role of the CP during immune cell invasion into the CNS following brain infection can significantly contribute to the damage caused by the disease. In surviving individuals this damage can manifest in long term neurological sequelae like hearing loss (Hahn et al., 2005). Rare cases of cryptococcal invasion into the CP have been described, which can lead to choroid plexitis. As consequences of this plexitis hydrocephalus and brainstem compression have been observed in patients with cryptococcal CNS disease (Graciela Agar et al., 2009). During Enteroviral meningitis, infection with CVB3 can cause severe pathology and apoptosis in the CP of mice (Tabor-Godwin et al., 2010; Puccini et al., 2014). It has been hypothesized that hydrocephalus, which occurred in mice infected with CVB3, could be related to dysfunctions of the CP caused by cell death (Rhoades et al., 2011). Hydrocephalus together with ventriculitis has also been described as complication for cases of *L. monocytogenes* brain infection (Ito et al., 2008; Ben Shimol et al., 2012). More research is required to clarify the role of the CP in neurological complications arising from CNS infection.

## The Choroid Plexus as a Target for Treatment?

Although the *plexus choroideus* has been neglected as a potential target for therapy in the past, more recently the putative therapeutic potential of the CP has come into perspective (Dragunow, 2013; Lehtinen et al., 2013). Attempts being made include strategies to design peptides, which allow targeting of the CP for delivery of compounds to the CNS, or the production of therapeutic proteins or peptides by genetically engineered stem cell derived CP epithelial cells (Gonzalez et al., 2011; Watanabe et al., 2012). Due to its extended involvement in the pathogenesis of brain infections the CP could be also considered as an interesting target for treatment of CNS infectious diseases. An important consideration would be evaluation of the potential of candidate drugs to cross the BCSFB for treatment in the CSF. As an example, a primary BCSFB model based on rat CP epithelial cells has been used to evaluate different nucleoside drugs for their transfer properties specifically across the blood–CSF interface (Strazielle et al., 2003).

Treatment of CNS infections includes adjunctive therapies like dexamethasone, but the use of dexamethasone in patients with proven or suspected meningitis has not been uniformly beneficial (Brouwer et al., 2013). In an *in vitro* model using PCPEC infected with *S. suis*, dexamethasone prevented alteration of TJ-associated proteins and barrier function (Tenenbaum et al., 2008), but the *in vivo* relevance of these findings remains to be determined. Manipulation of CP barrier function could in turn influence CNS entry of leukocytes across the CP, which is a main route for immune cells during inflammatory events in the brain (Engelhardt and Sorokin, 2009; Schwartz and Baruch, 2014). This is of special importance, since the migration of immune cells into the brain can be either helpful or deleterious during CNS pathologies (Schwartz and Raposo, 2014). Further research involving the use of functional *in vitro* models of the BCSFB will be required to investigate the suitability of the CP as therapeutic target.

## Concluding Remarks

Extensive research has shown that the CP plays multiple roles during infectious diseases of the CNS. These roles include the function as an entry site of the pathogens into the CNS as well as active participation by producing cytokines and chemokines and enabling the recruitment of immune cells. However, important details, particularly concerning the role of the CP in complications arising from CNS infection, still have to be discovered. Furthermore, it has to be answered whether the CP can serve as a target for treatment of CNS infection. In any case, functional *in vitro* models of the BCSFB will be of help to identify drug candidates with the potential to overcome the BCSFB and with the promise to interfere with disease progress and outcome.

## Conflict of Interest Statement

The authors declare that the research was conducted in the absence of any commercial or financial relationships that could be construed as a potential conflict of interest.
